# Supplementary material for: Image processing improvements afford second-generation handheld optoacoustic imaging of breast cancer patients
Source: Photoacoustics. 2022 Mar 2;26:100343. doi: 10.1016/j.pacs.2022.100343 (PMC8931444; doi:10.1016/j.pacs.2022.100343)
Supplement: Supplementary file 1 — Supplementary material [file mmc1.docx]

**
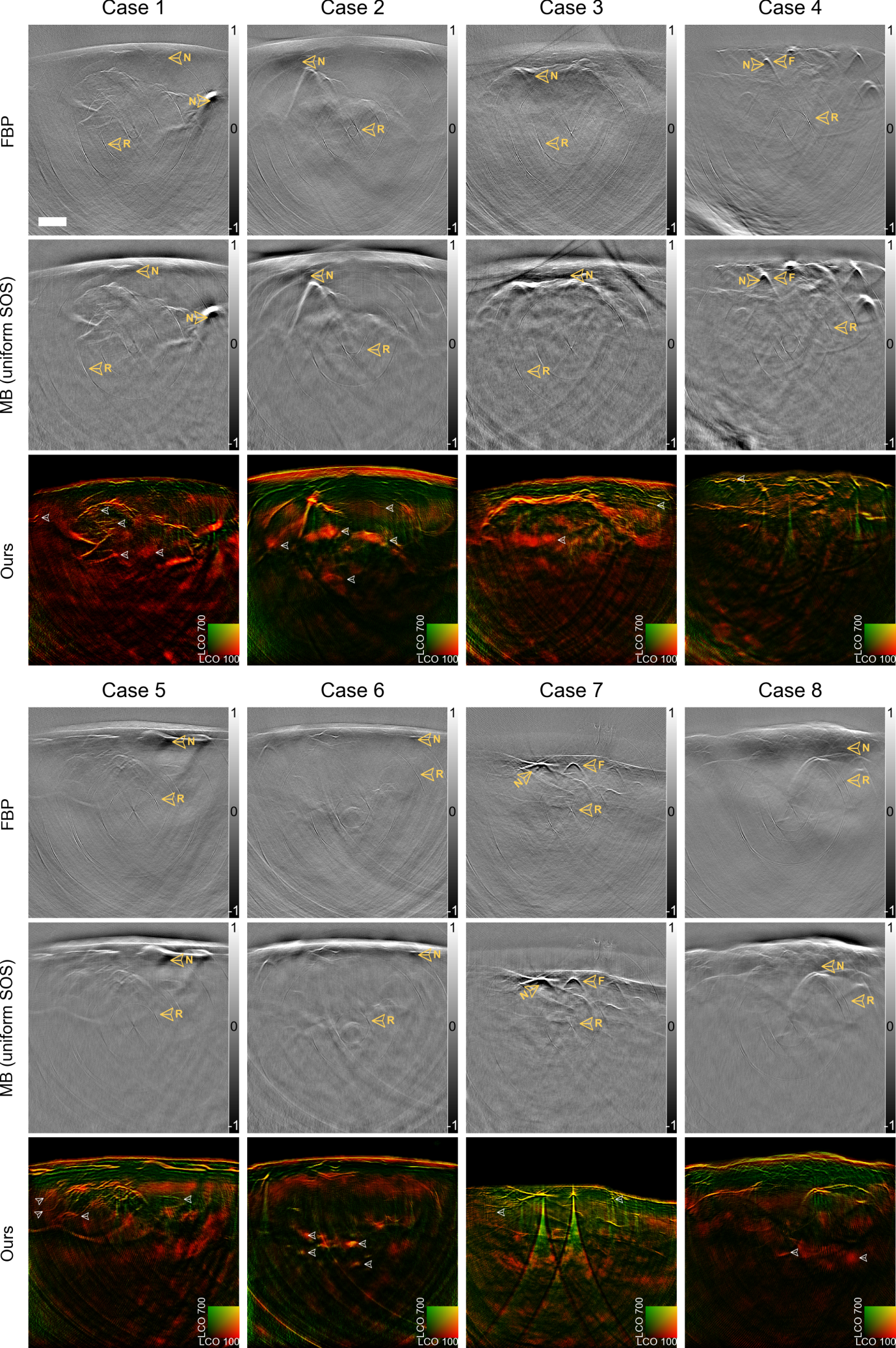
**

**Supplementary figure S1. Visual comparison of images reconstructed using filtered back-projection (FBP), simple model-based inversion (MB), and our proposed pipeline with an improved model.** Yellow arrows in the images denote some examples of image quality issues present in the FBP and simple MB reconstructions: negative values (N), bad focus (F), and ring-shaped noise artifacts (R). Furthermore, white arrows in our proposed images denote examples of fine details that cannot be properly distinguished in the images using FBP and simple MB. These include smaller blood vessels and deeper structures.

**
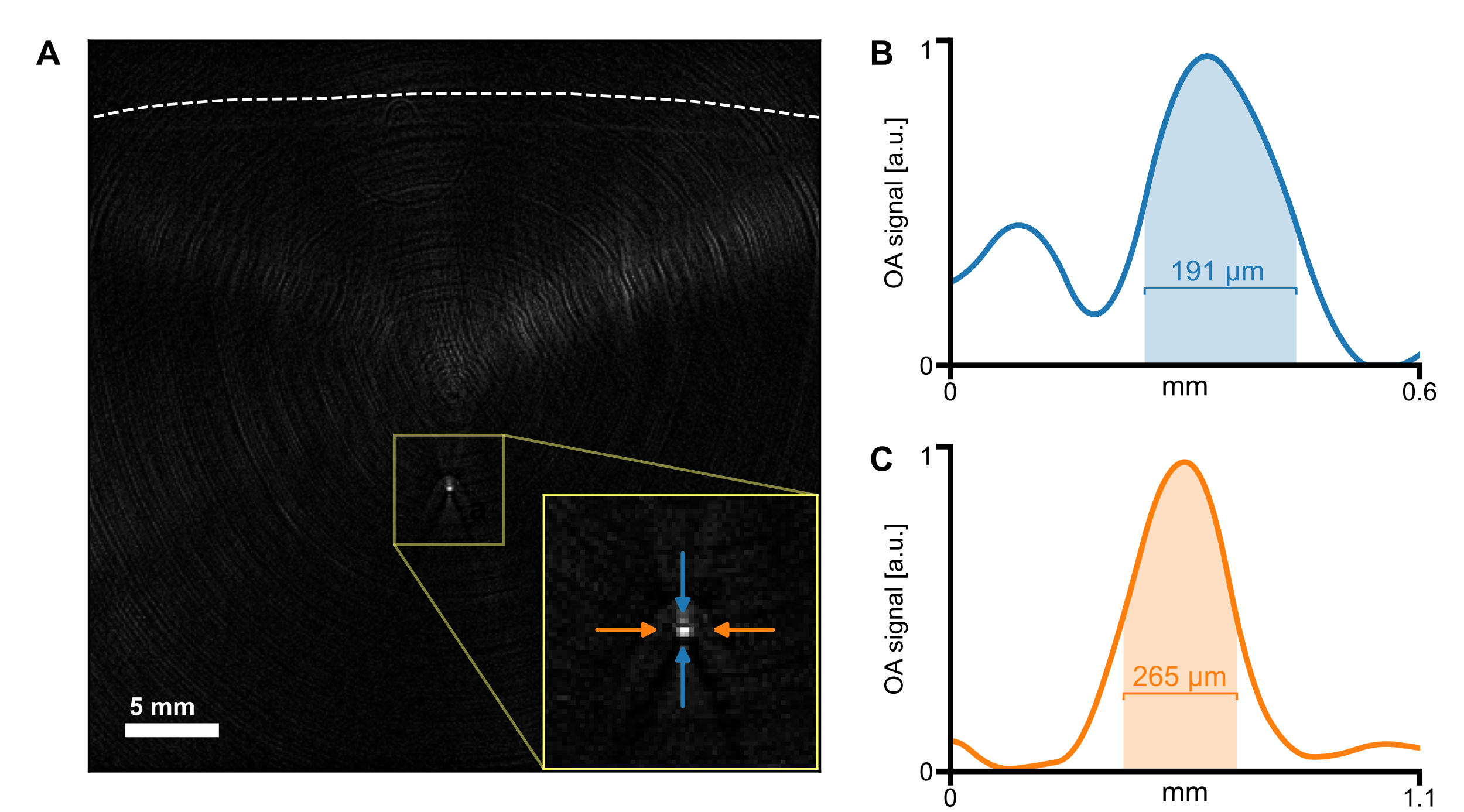
**

**Supplementary figure S2.** Phantom measurement of the attained resolution. A, A 100 μm microsphere was embedded into an agar cylinder and placed in water bath ca. 2 cm below the probe membrane (denoted by the dashed line). The figure shows the image reconstructed from mean signal of 19 MSOT frames using our model with total impulse response correction and spectral median taken over the wavelength range 780–820 nm. A detail of the image containing the microsphere is shown in the inset magnification. Blue and orange arrows mark the ends of axial and lateral line profiles, respectively, which are plotted in panels B and C. B, Axial line profile of the imaged microsphere with 191 μm full width at half-maximum (FWHM). C, Lateral line profile of the imaged microsphere with 265 μm FWHM.

**
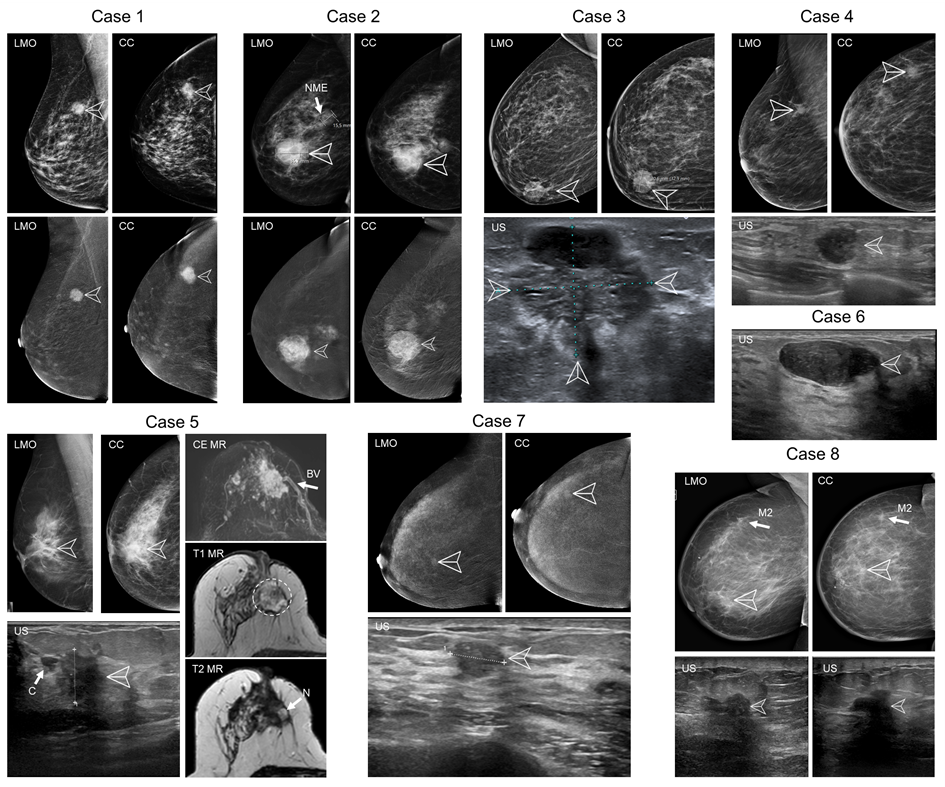
**

**Supplementary figure S3:** **Conventional clinical images of the eight cases presented in the results.** Lesions are marked by arrowheads. **Case 1:** Contrast-enhanced mammography of the right breast showing the enhancing mass with indistinct borders in the upper outer quadrant in lateromedial oblique (LMO) and craniocaudal (CC) views. **Case 2:** Contrast-enhanced mammography (LMO and CC) of the right breast showing a mainly circumscribed, partially indistinct tumor and adjacent non-mass enhancement (arrow NME) without clear findings in the Low energy (upper) image. **Case 3:** Mammography (LMO and CC) and a conventional ultrasound (US) image of the tumor containing well circumscribed hypoechoic parts (either cystic or containing more aggressively growing cells) and some spiculated, less hypoechoic infiltrating parts. This example reflects tumor heterogeneity. **Case 4:** Mammographs (LMO and CC) and a conventional ultrasound image of the microlobulated round tumor. **Case 5:** Mammographs (LMO and CC), a conventional ultrasound, contrast-enhanced, T1- and unenhanced T2-weighted magnetic resonance (MR) images of the partially necrotic tumor. A large feeding blood vessel subtracted CE-MR, arrow BV) is visible near the lesion in a position matching the observation in the OA image (Fig. 4F, arrow 1). Necrosis is visible in the tumor core (T2 image, arrow N) usually reflecting a very fast tumor growth. The ultrasound shows a cyst or a dilated duct in a close proximity to the tumor core (arrow C). **Case 6:** An ultrasound image of a fibroadenoma. The lesion has a typical appearance: hypoechoic, oval in shape, with a uniformly distributed texture and smooth borders. **Case 7:** Contrast-enhanced mammography (LMO and CC) and an ultrasound image of the breast containing the tumor. The 10 mm large lesion is oval to round, with unsharp borders and a disruption of some anatomical structures. **Case 8:** Mammography (LMO and CC) and ultrasound images of the breast with a recurring inflammatory mamma carcinoma with skin thickening and two masses: arrowheads mark the mass imaged in this study, appearing in the as irregular mass with shadowing features. A secondary mass is marked by arrow M2.
